# Are researchers getting the terms used to denote different types of recreational cannabis right?—a user perspective

**DOI:** 10.1186/s42238-021-00065-1

**Published:** 2021-04-29

**Authors:** Ava Mason, Musa Sami, Caitlin Notley, Sagnik Bhattacharyya

**Affiliations:** 1 Department of Psychosis Studies, Institute of Psychiatry, Psychology and Neuroscience, King’s College London, London, SE5 8AF UK; 2 Institute of Mental Health, University of Nottingham, Triumph Road, Jubilee Campus, Nottingham, UK; 3 School of Medicine Health Policy & Practice, University of East Anglia, Norwich, UK

**Keywords:** Cannabis; Cannabis terms; Skunk; Cannabis users; Iterative categorisation

## Abstract

**Background:**

While current cannabis research has advanced our understanding into the effects of its individual components, there is a pressing need to identify simple terminology that is understood in the same way by researchers and users of cannabis. Current categorisation in research focuses on the two main cannabinoids: delta-9-tetrahydrocannabinol (THC) and cannabidiol (CBD); and two different species of cannabis: indica and sativa. Recreational cannabis has also been categorised by researchers as ‘skunk’ or ‘hash’. Focusing on individuals who use cannabis frequently, this study aimed to identify views on current terms used to denote different types of cannabis and to identify terms validated by participants. These views were extracted from responses of the Cannabis Experiences Questionnaire (CEQ), a widely used instrument in the literature.

**Methods:**

We qualitatively analysed 236 free-text responses from Question 23 of the CEQ survey (using Iterative Categorisation) relating to categorization and consumption methods. Data was used from a previous study (Sami et al., Psychol Med 49:103–12, 2019), which recruited a convenience sample of 1231 participants aged 18 years and above who had previously used cannabis.

**Results:**

Regarding type of cannabis used, specific strain names (*n* = 130), concentrates (*n* = 37), indica/sativa (*n* = 22) and THC/CBD terms (*n* = 22) were mentioned. Other terms used were hybrids (*n* = 10), origins of specific strains (*n* = 17), edibles (*n* = 8), and herbal cannabis (*n* = 7). Regarding problems with specific terms, participants were skeptical about terms such as skunk and super skunk (*n* = 78) preferring terms like THC/CBD, indica/sativa, specific marketed strains and references to preparation methods.

**Conclusions:**

The results suggest a disparity between the common terms used by researchers in academia and those used by cannabis consumers. While there are advantages and limitations of using these terms to bridge views of researchers and individuals who use cannabis, this study underscores the importance of formally assessing chemical constituents rather than relying on self-report data and of incorporating cannabis user views on current terms used in research, potentially also incorporating descriptors of preparation and consumption methods.

**Supplementary Information:**

The online version contains supplementary material available at 10.1186/s42238-021-00065-1.

## Background

Cannabis is one of the most widely cultivated psychoactive drugs consumed by 3.9% of the global population aged 15–64 years old World Drug Report (2020). Cannabis has over 120 different cannabinoid compounds (specific to cannabis) with over 600 different terpenoids and flavonoids (Ahmed et al. 2015; Radwan et al. 2015). The two primary cannabinoids that are most abundant in common cannabis strains are: delta-9-tetrahydrocannabinol (THC) and cannabidiol (CBD). Human experimental studies have shown that acutely both THC and CBD may have opposing effects on behaviour and cognition (Bhattacharyya et al. 2010; Colizzi and Bhattacharyya 2017; Hindocha et al. 2015). Following chronic exposure or repeated dosing, THC may increase the risk of onset and relapse of psychosis, while CBD may oppose its harmful effects (Morgan and Curran 2008; Schoeler et al. 2016) and have antipsychotic effects (Schoevers et al. 2020) with good tolerability (Iffland and Grotenhermen 2017).

Due to the opposing health effects seen with different cannabinoids, recent research has moved from treating cannabis as one entity to a more fine-grained understanding. This includes measuring the concentration of cannabinoids, particularly THC and CBD (Englund et al. 2017; Morgan and Curran 2008). Botanists have combined this research of cannabinoids with their focus on morphological distinctions (Clarke and Merlin 2016) to categorise cannabis types into two species: sativa and indica (Piomelli and Russo 2016). Cannabis sativa often has lower levels of THC to CBD, compared to cannabis indica, presenting with higher levels of THC (Clarke and Merlin 2016; Hillig and Mahlberg 2004). It is possible to crossbreed cannabis plants to contain both indica and sativa characteristics, producing strains known as hybrids, potentially affecting the validity of such categorization (Piomelli and Russo 2016). Market strain names (such as ‘Haze’) that are bought by individuals who recreationally use cannabis are usually described based on their species and THC content. As well as these more detailed outlooks, research has also focused on how the method of preparation and consumption alters the effects of cannabis. Methods such as ‘vaping’ (Budney et al. 2015) and ‘blasting’, where products such as ‘shatter’, ‘wax’ or ‘dabs’ are consumed can cause products originating from the same plant to often have dissimilar effects (Stogner and Miller 2015a) due to variability in the amount of THC and the rate at which it may be consumed through different methods (Stogner and Miller 2015b). Therefore, how cannabis is consumed may also influence its effects apart from the concentration of different cannabinoids.

Self-report measurements have been used to examine detailed responses on the type of cannabis used by participants (The Medical Marijuana Patient Use Questionnaire, Bonn-Miller et al. 2014; Cannabis Experience Questionnaire, (Barkus et al. 2006) and the motives underlying use (Connor et al. 2011). The Cannabis Experience Questionnaire, on which several findings have been based (CEQ, Di Forti et al. 2009; Di Forti et al. 2015; Sami et al. 2019; Schoeler et al. 2016; Quinn et al. 2017) uses the terminology resin/dried plant, herbal cannabis (outdoor grown) and sinsimilla/skunk. Skunk is a non-pollinated seedless cannabis plant (Freeman and Winstock 2015), grown indoors in highly controlled conditions (Potter et al. 2008), and high in THC (THC at 1–23%; Potter et al. 2008). Herbal cannabis also contains high THC levels (9%; Freeman and Winstock 2015) and low CBD (below 0.1%; Potter et al. 2008).

However, terminology used in research reports often neglect terms like indica/sativa, THC/CBD or description of the specific preparation and/or consumption methods when asking users of cannabis about their drug-induced experiences. Furthermore, it remains unclear whether terms such as ‘skunk’ often used in research focusing on police seizures of cannabis (Englund et al. 2017) are used by recreational cannabis users themselves to describe the specific type of cannabis that they may seek for use. As self-report data is often relied upon to understand the psychological effects of drug-induced experiences (Hardwick and King 2008; Potter et al. 2008) in the content of cannabis use, it is particularly important to align terminology, such that researchers and individuals who use cannabis employ terms that have the same meaning or refer to the same thing. In the absence of a common set of terms used to describe different types of cannabis by both researchers and people who use cannabis, respondents of questionnaires describing experiences associated with the use of different types of cannabis use may be referring to something completely different from how researchers interpret their responses, affecting the usefulness and validity of much painstaking work.

Therefore, the aims of this exploratory study were to aid in the development of common language to describe cannabis related terms used by both the cannabis user and researcher, increasing methodological validity of future cannabis research. Cannabis terminology in this study relates to terms used to describe different types of cannabis products and not legal categories as described under the Misuse of Drugs Act 1971.The objectives were (1) to identify views of experienced users of cannabis on current terms used to describe different types of cannabis used recreationally and (2) to identify terms that were validated by the respondents. These aims were achieved by qualitatively analysing the responses from the Cannabis Experience Questionnaire, focusing on consumption method, type of cannabis smoked and reasons for consuming their choice.

## Methods

Following ethical approval from the Kings College, London Research Ethics Committee (REMAS HR-14/15-0551), a web-based survey based on a modified version of the Cannabis Experiences Questionnaire (Barkus et al. 2006) was carried out. Participants were given an information sheet online and were asked to sign an online consent for (as required by the ethics committee) before they were able to take part in the survey. The survey was available to members of the public aged 18 and over, and data was collected between December 2015 and September 2016. Participants were recruited through advertising from the King’s College London mailing list, as well as through social media sites (like Facebook, Tumblr and Twitter), given the opportunity to enter into a raffle of three prizes worth £10–£50 on completion of the questionnaire. Requests were made through social media with cannabis campaigning organisations, including the London Cannabis Club and CLEARUK to promote the study. The questionnaire was primarily used to collect data on cannabis use. We planned to address the main objectives once the data collection has ended. Using data collected from this survey, we have previously examined the association between psychotic-like experiences associated with cannabis use and cessation of cannabis use in a previous manuscript (Sami et al. 2019). The present study focuses on previously unreported data from the survey, specifically questions regarding the method of cannabis consumed, type and reason for this.

### CEQ questionnaire

The survey was based upon an instrument developed in Manchester and Wollagong (Barkus et al. 2006; Barkus and Lewis 2008) finding widespread use by researchers across populations over the last decade (Barkus and Lewis 2008; Bianconi et al. 2016; Quinn et al. 2017). This questionnaire is a 46-item self-report scale focusing on frequency/pattern of use, age and type of use. The experience subscales are divided into pleasurable, psychosis-like and after-effect experiences (Barkus and Lewis 2008).

The survey used mixed methods including Likert scales for cannabis experiences and categorical responses to determine demographic data. Regarding the type and method of cannabis consumed, the survey asked the following question:

(i) What type of cannabis do you use the most:
  Hash (cannabis resin/solid)
  Imported herbal cannabis
  Homegrown skunk (sinsimilla)
  Superskunk
  Synthetic cannabis (such as spice or black mamba)
  Other (please specify) if there are names of brands please also specify: (free-text)

### Qualitative analysis method

Qualitative analysis of all free-text responses was undertaken by a postgraduate psychology student (AM) supervised by qualitative methodologist (CN). These responses were in the free-text response mentioned earlier; the ‘other’ option regarding cannabis type consumed. Methodology followed was that of Iterative categorisation (Neale 2016), a systematic approach combining deductive and inductive coding specifically developed for analysis of qualitative data within the addictions research field. Deductive codes were developed through team discussion a priori, supplemented by inductive generation of ‘in vivo’ codes derived from the data (Neale 2016).

Prior to analysis, a deductive coding data file was developed to incorporate all responses. Inductive coding was generated, working line by line through the content of each free-text response. Responses were categorised by type of cannabis consumed views of respondents on current terms, terms validated by individuals who use cannabis and information about the marketed strain sources. The second step involved constructing a coding file, where the inductively derived codes were matched to the deductive coding file initially created. This was then condensed to an ‘enablers’ file (Additional file 1: Appendix 1), where similar responses were grouped together per coding category. Lastly, a summarised analysis file (Additional file 1: Appendix 2) was created to interpret the responses from each category. Analysis was discussed and agreed as a team throughout the process.

## Results

### Demographics

There were 1425 individuals who responded to the survey, including responses from 1231 individuals who had used cannabis at least once and 926 current cannabis users. One thousand one hundred seven participants provided any form of qualitative answers, i.e., they filled in any of the free-text boxes related to the reasons for their cannabis use or changes in use. Two hundred thirty-six (16.5%) participants provided qualitative answers for the CEQ question “What type of cannabis do you use the most” in the ‘other’ free-text response.

68.7% of respondents were male with an average age of 29.5 years (s.d. 10.3 years). Although the country of respondents was not routinely asked during the study, 531 participants agreed to a follow-up study and gave their place of residence. Four hundred ninety-four (93%) participants lived in the UK and 23 (4.3%) from Sweden. There were also a few responses from Brazil, Greece, Mauritius, the USA and Zimbabwe. 38.1% of the respondents reported previous mental health contact. While the free-text information did not quantify this, it appeared to be related to depression, anxiety or stressful experience and treatment by counselling or a primary care setting. Regarding cannabis use, mean age of first use was 16.7 years (ranging from 7 to 55 years old). 75.2% of participants continued to use cannabis, with 18.4% agreeing they would stop in the future. Regarding frequency of cannabis use, 44.4% of participants used cannabis every day, 22% used it more than once a week, 13.2% used it a few times a month, 10.7% a few times a year and 5.6% once or twice generally. 4.1% of the data was missing for this question.

### Quantitative analysis of response frequency

From 236 free-text responses regarding type of cannabis used most commonly, 21 different themes/terms emerged as mentioned in the comments (350 total references, as some responses mentioned more than one theme). Regarding type of cannabis used, specific strain names were mentioned most frequently (130 out of 350 references), the most popular being Haze (46 out of 350), Cheese (27 out of 350) and Kush (19 out of 350). Other common cannabis types referred to were concentrates (37 out of 350; including ‘concentrate’, resin, dabs, wax, BHO/butane and shatter), indica/sativa (22 out of 350) and THC/CBD terms (22 out of 35). Terms such as hybrids (10 out of 350), strain origins (17 out of 350), edibles (8 out of 350) and herbal cannabis (7 out of 350) were mentioned less frequently. Out of all the participants (*n* = 236) who filled out free-text responses via the text box, 98 participants expressed dissent about the terms used by researchers to commonly describe types/categories of cannabis in their free-text responses regarding type of cannabis used. Participants most commonly expressed concern with use of the terms skunk and superskunk (78 out of 350) to describe cannabis categories. Less frequently, concern was expressed about the terms sinsemilla (7 out of 350), synthetic (5 out of 350), imported (5 out of 350) and homegrown (3 out of 350) used to describe cannabis types.

### Qualitative analysis

Two over-arching themes were examined from the qualitative analysis: (1) views of respondents on current terms and (2) terms validated by experienced cannabis users. Regarding the first theme, terms that respondents commented on were sinsemilla, homegrown, synthetic, imported and skunk/super skunk. However, a significant amount of responses related solely to the terms skunk/super skunk. Focusing on these terms, responses suggested that these terms were (i) unknown and undefined, (ii) incorrectly applied terms from the media, (iii) used to describe a wide range of market strain names and (iv) outdated terms referring to high THC levels.

Responses relating to the second theme ‘user-validated terms’ were categorised into (i) THC and CBD (ii) indica/sativa. (iii) Popular marketed strains and (iv) different preparation methods.

#### Theme 1: views of respondents on the current terms; skunk/superskunk

##### (1i) Terms are unknown and not clearly defined

Participants reported these terms as not clearly defined or well-known terms. As many of these participants considered themselves to be frequent users of cannabis, they seemed certain that these terms were not in common use amongst cannabis users or that their existence was a ‘myth’. Example responses from participants in this category were the following:

- ‘What is homegrown skunk or super skunk- need to get terminology right before publishing.’
- ‘What is super skunk? This terminology is unfamiliar to me - and I talk to a lot of cannabis users it really should be familiar.’
- ‘I smoke strong home grown weed, there is no such thing as “skunk” or “super skunk” in the real world.’
- ‘Don't entirely understand the names given above. I mostly smoke marijuana (skunk?), either home grown by my friends and/or dealers or imported from abroad. Not sure what “super skunk” is.’

##### (1ii) These terms are often used in the media, not scientific surveys

One of the comments paired with the terms being unrecognised was their use in the media. Specifically, ‘skunk’ was seen as unacceptable and more a ‘buzzword’ that should not be used in a scientific setting. Some responses stated that the media use this word to signify ‘well grown cannabis plants’ or ‘the flower of the plant’. While all these responses suggested a negative view on media use, some went on further to say that use of these ‘buzzwords’ causes individuals who use cannabis to lean heavily away from these terms. These responses can be seen below:

- ‘Don’t know what you mean by skunk/super skunk. If you mean do I smoke the flower of the plant then yes but that doesn’t necessarily mean that its skunk. Not all cannabis flowers are skunk. Maybe do some research instead of using terminology used in the media. Especially if you’re going to run a survey’.
- ‘Amongst ‘experienced’ users, ‘skunk’ is not a recognised term-there is not a consensus on what exactly it is and the media over-using it as a buzzword causes us to lean heavily away from the term.’
- ‘By ‘super skunk’ I assume you’re just referring to what the media call skunk, aka well grown cannabis plants..?’
- ‘Inadequate- super skunk/skunk not acceptable terms- used solely by the media to promote cannabis.’

##### (1iii) These terms rarely reported by consumers are being used to generalise a wide range of market strain names

Participants suggested that using the term skunk portrayed a lack of awareness of different marketed strains that were available. Responses also stated that this specific strain should not be highlighted as a main categorisation method, as it is not the main strain consumed by individuals who frequently use cannabis. This suggested perhaps a lack of understanding of the complexity of strains available and need on the part of researchers to reflect that in terms used to denote different types of cannabis strains available for recreational use. Examples of these responses can be seen below:

- “There are a thousand and one strains available. Skunk is one of them. It's like calling your vacuum cleaner a Hoover even if it’s made by Dyson.”
- ‘Skunk is a specific strain, not a “type of cannabis”. This is like asking “what type of alcohol do you use mostly? Vodka or Gin”. This question is fundamentally flawed for the information you are trying to get.’
- ‘I'm sorry: The terminology you have used makes it impossible to answer the question. “Skunk” is a single strain or small grouping of strains only, when there are 100s od strains that are not “Skunk”.

##### (1iv) Overused and outdated term referring to THC levels

Respondents also stated that the terms skunk and super skunk were often paired with THC levels that could be seen as terminology method in itself. Responses stated this was invalid as skunk itself is not strong and may not have significantly higher THC levels compared to other strains. This can be seen in the below responses:

- ‘Firstly, most dealers don’t have a clue whether the cannabis was imported or home grown and fewer tell the customer. Secondly, what on earth is super skunk? If you do a little bit of reading and see how cannabis is bred you will know skunk as you call it doesn’t exist! That name being applied to high THC cannabis is plain wrong’
- ‘Skunk and super skunk I find derogatory terms used to describe cannabis with low cbd content and a higher thc content. It’s like describing a bottle of chateau rothschild like a bottle of lambrini. I use none of the above but I do use a good quality cannabis not from the street’.
- ‘I can only assume you are using the terribly inaccurate and misleading media term of cannabis that contains very high levels of THC and very low levels of CBD. Skunk itself it actually not that strong at all.’

#### Theme 2: terminology mentioned by the respondents

##### (2i) THC and CBD

One alternative method of categorisation that was suggested seemed to be based on THC/CBD ratio. Conflicting responses were found referring to the ratio preferences of THC:CBD, with some showing no preference or favouring a 1:1 ratio. Other participants specified a preference for higher CBD to THC, contrasting responses preferring high sativa skunk with higher THC levels.

This was also connected to the method of preparation and consumption, with responses stating that cannabis oil has a ‘better balance of CBD to THC’, mentioning its use for THC wax (concentrated THC smoked off a vaporising pin) and use in pill form (500 mg CBD). Market strain names and indica/sativa terms were also paired with THC/CBD terms, suggesting a link to other terms that could also be used as valid terminology.

One participant stated that people who use cannabis needed to be responsible enough to know the difference between use and abuse when using high THC marketed strains. This suggests the importance of the actual approach and use of cannabis in relation to the chemical constituents consumed. While there were differing views on THC:CBD ratio preference and its paired terms, it was seen by participants as a means of identifying and categorising cannabis strains.

- ‘1:1 ratio THC/CBD Indica strains’.
- ‘The weed I buy is mostly sativa but now and again I go for an indica. It probably has a fairly high THC/CBD ratio as the buds a well formed and have a good coating of trichomes’.
- ‘Girl scout cookies indica over 20% thc low cbd. Pineapple express over 22% thc’.
- ‘High sativa skunk preferred as has good THC level.’
- Consumes cannabis oil- THC wax, concentrated THC smoked off a vaporising pin [p28].
- ‘I prefer less of the THC and more of the CBD in a strain that is what seems to give me the most effective medicinal/pain relieving properties’.
- ‘Likes other strong/high THC strains you need to be responsible to know the difference between use and abuse’.

##### (2ii) Indica/sativa

Another term raised almost as frequently as THC/CBD was indica and sativa. When mentioned, general preference was usually for indica, with both indica and sativa stated as helpful for medical purposes. Like THC/CBD, this phrase was often paired with a marketed strain term. Additionally, hybrids (a mixture of sativa and indica variants) were often mentioned. These responses (shown below) point towards a view that cannabis plant species may be considered by individuals who use cannabis as a viable categorization method.

- ‘There are two different types of cannabis: indica and sativa’.
- ‘There are many different cannabis strains eg. Blue Cheese, Afghan Kush, Silver haze. There is also two different types of cannabis, indica and sativa. The tick boxes are not adequate’.
- ‘I normally try to find an indica that’s got a good amount of CBD. I have back pain and constant sciatica. It's excellent as a muscle relaxant and helps as a sleep aid’.
- ‘Sativa heavy hybrids are what they like the most, such as Jack Herer perfect for medical and recreational purposes’.
- ‘Uses Jack Herer- an indica dominant hybrid made by combining shiva skunk (indica) and northern lights (indica)’.

##### (2iii) Popular marketed strains

Instead of terms like skunk and THC/CBD used in addiction research, marketed strains were most frequently mentioned in the free-text response of cannabis type used. Frequently mentioned strain names were various Hazes, Kush and Cheese strains. In this sample, Lemon haze was the most frequently mentioned strain consumed, followed by amnesia haze, super silver haze and other hazes; grapefruit, Abyssinia, mango, silver haze, super bubblegum and golden haze. In terms of strength, participants mentioned general haze to be far stronger than other strains such as super Skunk, specifically mentioning super silver haze and super lemon haze.

Consumption of cheese strains was another frequently mentioned response, with participants stating it was mainly common in England and seen as highly regarded to get a ‘high’. Different cheeses mentioned were Blue (seen as the strongest), Dutch and Buddha cheese. Six participants consumed Jack Herer, (described as an indica dominant hybrid), combining Shiva skunk with Northern lights indica strain. Jack Herer was utilised for medical (particularly pain-relief) and recreational purposes and helps with energy levels. The frequency of responses relating to each strain name can be found in Additional file 1: Appendix 3.

##### (2iv) Alternative preparations concentrates/oils/edibles

Vaping and dabbing (formerly mentioned in the introduction) were noted in the free-text response to be newer methods of consumption not included in the current survey, with other excluded options being consuming shatter, crumble and wax. A variety of oils were mentioned for example chocolate infused hash oil and various CBD oils. Edibles mentioned included infused coconut oil (seen as an appetite stimulant), butter weed with coffee (seen as relaxing), cannabis butter with already vaped herbs and hash infused chocolate. Considering the wide range of consumption methods as well as the effect these may have on the user’s experience suggest the need to add these newer methods to current terminology. The range of responses can be seen below:

- ‘Question is idiotic and misinformed, as are a few of the others already answered (no option for vaporising/dabbing in method of consumption question)’.
- ‘The survey is incomplete- either vape medical weed or concentrates, oil, shatter, wax in vaporiser not smoking at all’.
- ‘Butter weed with coffee= relaxing’.
- ‘Wax, shatter and concentrates are also widely used, but too much money (£40-100 per gram) too strong for me and builds dependency and tolerance levels’.
- ‘Then I make cannabis butter with the already vaped herbs and coconut oil, this makes a great muscle relaxant, painkiller, anti-depressant and appetite stimulant’.
- ‘Not quite sure what type of cannabis I smoke- almost all through a vape pen heating up cannabis oil which is then inhaled better than inhaling smoke from flames’.

## Discussion

In this exploratory study, we analysed qualitative responses of individuals who were experienced users of cannabis with regard to their views on terms used in the academic setting to describe cannabis. The results may suggest a potential disconnect between the common terms used by researchers to denote different types of cannabis and those used by cannabis consumers. Regarding the first aim, a sizeable proportion (around 40%) of those providing free-text responses indicated disagreement with the common terms used in research contexts to denote various types of cannabis. To a large extent, this disagreement was about use of the terms ‘skunk’ or ‘superskunk’. Views were that these terms were seen as incorrectly applied to cannabis with high THC levels and a wide range of marketed strain names in both the research setting and in media reports. Secondly, terms validated by respondents that were also used in research were THC/CBD and indica/sativa. Other terms that were also deemed as important to respondents were of marketed strain names, e.g. ‘haze’ and terms that referred to different methods of preparation of cannabis for use. This underscores the importance of involving cannabis users in the development of assessments for assessing cannabis use and its effects. Common terms used by both researchers and individuals who use cannabis often could include focusing specifically on THC/CBD and indica/sativa terms and always including a question on the preparation method used.

When considering the strong opinions raised towards the term ‘skunk’, it is important to review the contextual history of its use. Potter and Chatwin (2012) have stated that the term ‘skunk’ originally had a precise meaning, relating to indoor-grown, high potency marketed strain. It has now been established in public discourse, in the media and by policy makers and academics to encompass a broad definition of a larger cannabis subset. Many participants in the present study indicated that the term generalises to a wide range of marketed strain names and should not be used as one of the key terms to categorise different types of cannabis. This is consistent with the idea that the term may convey different meanings to different individuals and may generalise a specific marketed strain to also refer to adulterated cannabis. Two other key themes raised by respondents suggested that skunk is often paired with high THC levels and is an overused term used in the media. This mirrors comments by Stevens (2007) that the term has often been used in a pejorative sense to indicate excessive use of high potency cannabis. Responses in the present survey also seem to mirror participants’ responses obtained in the survey carried out by Potter and Chatwin (2012) as part of their qualitative analysis on cannabis classification. The views generated by the term ‘skunk’ perhaps may also indicate a distrust in the mind of some cannabis users that views of researchers about cannabis may potentially be coloured by prejudice. This may be due to the term inadvertently reinforcing existing media myths as well as due to the lack of a shared precise term meaning. Therefore, it may be the case that excessive use of this once precise term may have caused its original meaning to be lost and increased distrust in people who use cannabis frequently. This may have a general negative effect on participants who may be put off by research simply due to the use of terms like ‘skunk’, underscoring the importance of public involvement in understanding cannabis terminology to ensure that terms used are deemed credible by both people who use cannabis and researchers. However, it is also important to put this in perspective in that a relatively modest proportion of participants in the survey provided free-text responses regarding type of cannabis used, of whom less than half expressed negative views about the term ‘skunk’.

Relating to the second aim, terms mentioned by respondents that were deemed as credible were those based on THC/CBD ratio and indica/sativa strains. Both these terms are used by researchers and botanists (Hillig and Mahlberg 2004; Holland and Torres 2019), and suggest the importance of reporting THC and CBD concentrations within the field of cannabis research. While these responses may suggest that these are valid methods of categorization, there are issues with the terms indica/sativa. In particular, considerable interbreeding and hybridization may have rendered any attempts at categorization based on physical properties of the plant (such as branching, height, leaf morphology) prone to errors and of limited utility in the absence of biochemical assays (Piomelli and Russo 2016). Other terminology highlighted by respondents was the manner in which cannabis was prepared and consumed. Examples of this would be vaping, dabbing, edibles through infused oils, coffee, butter and chocolate. Method of consumption is particularly important when considering how cannabis with high THC concentration may be prepared and consumed in a specific way by cannabis users (Kilmer et al. 2013). This makes it harder to compare results from different academic studies or generalise these results to cannabis use in the general population when consumption methods may differ. Referencing consumption method also is useful in that, while participants may be unaware of the THC levels or specific strain consumed, they will have direct knowledge of the way they consume it. Therefore, including description of the consumption method in research could increase the validity of research particularly when generalising such results to the wider cannabis user population.

Terms mentioned by respondents that are not often used in research were specific marketed strain names. While internet databases often describe psychological characteristics and biochemical measurements of specific marketed strains (Leafly 2020), these terms are rarely used by academics. The reason for this may relate to all the variables that could interfere with the results’ validity. Each strain comes with a wide range of subjective experiences, perhaps due to different production companies selling seeds and strains between each other, affecting the characteristics of what is labelled as the same strain. Availability of strains is also susceptible to changes in consumer demand, thereby limiting the usefulness and reliability of marketed strain names over time as standard terminology. Also, specific strain names are inaccessible to researchers, meaning researchers would be relying solely on feedback from users. Englund et al. (2017) suggested that future methods of strain access could be to collect joints of specific strains from individuals who use cannabis and test their cannabinoid concentration. However, cannabis sample collection is expensive, yet to be validated and is challenging to do in certain geographical jurisdictions because of legal implications (e.g. in the USA). It is important that the method used is seen as unbiased to the cannabis user, reflective of actual usage as well as feasible to be applied by the academic and botanist. Therefore, use of marketed strain terms as a method of categorisation may have limited utility in the research context until such strains could be accessed for research.

In light of this, it is also important to consider the sample of participants who responded to this survey. Our sample reflected the views of more experienced cannabis users, who may have different opinions on terms used to refer to various types of cannabis used recreationally due to their greater knowledge and awareness of the effects of cannabis containing different combinations of cannabinoids. Individuals who use cannabis less frequently may have less familiarity with some of the terms suggested before (such as THC/CBD or indica/sativa). Therefore, in order to ensure appropriate use of such terms in the context of research on cannabis use, they may need to be tailored to target participants. Further, it is worth noting that out of over 1100 respondents who provided any free-text responses, only 236 participants provided responses regarding type of cannabis used, indicating that such views may not necessarily fully represent the views of the wider population of cannabis users. Additionally, it is important to highlight that most respondents of the present survey lived in the UK and therefore results presented herein may not reflect opinions of cannabis users residing in other countries. This may reflect the need for considering regional variation of terms used to refer to different types/ categories of cannabis.

The terms formerly mentioned that may be used to denote various types of cannabis with a precise meaning shared between researchers and users of cannabis present with their own limitations. Firstly, it is worth noting that previous work (Potter and Chatwin 2012) has indicated issues with use of the term ‘skunk’ as identified. As we used the CEQ, which had already been published and demonstrated high validity and reliability when administered in online, student, clinical and non-clinical cohorts (Barkus et al. 2006; Barkus and Lewis 2008; Stirling et al. 2008; Quinn et al. 2016), we did not carry out any separate piloting before initiating the present study. Nevertheless, results presented here highlight the importance of including potential respondents in the design of survey questions as well as piloting survey questions before they go live. Regarding the term ‘skunk’, research emerging out of certain countries such as the USA does not commonly refer to this term, meaning that concerns about use of this term may reflect more on UK-based research. Relating to indica/sativa terms, as highlighted before, focusing on the physical characteristics of the plant that help distinguish them may not reliably suggest the plant extract’s biochemical content (Piomelli and Russo 2016) and subsequent psychological effects. Previous research has suggested that the morphological distinctions between cannabis plants may be appropriate for cannabis plant growers interested in the botanic side (Jikomes 2018) rather than those investigating the psychological effects of different strains. Secondly, there is little evidence suggesting a concrete difference between indica and sativa, with recent evidence suggesting no distinct genetic difference (Schwabe and McGlaughlin 2019). Therefore, future research may focus on relating data from users’ feedback on specific effects with measurement of the actual THC and CBD concentration in cannabis used by them.

While the current study aimed to solely explore respondents’ feedback regarding cannabis terms, future work on cannabis experiences should not simply rely on self-report data. They should formally assess chemical composition of cannabis used and incorporate this with contextual information on the purpose of cannabis use (e.g. whether for ‘medicinal’ or recreational purposes). It is worth noting that other factors that may influence the effects of cannabis, such as origin of seed, storage or distribution methods were not considered in this study. This was due to the need to identify simple and understandable cannabis terms, rather than incorporate all factors (not raised by respondents) that may affect different components of cannabis. Additionally, data was not collected on where respondents were getting their cannabis from. This may have affected responses, as individuals buying cannabis online may be more likely to refer to specific branded strains, while categories used in research (skunk, oils or resin) may be more relevant to individuals who use dealers or friends. Therefore, future work should collect data on where participants’ obtain their cannabis from to help develop a fuller understanding. There is also a ‘self-selection’ bias, which may have affected the modest amount of free-text responses provided, with respondents being more likely to be current users compared to past cannabis users. Therefore, future studies looking at self-report data should consider recruitment strategies that ensure a more representative view of cannabis users and investigate differences between current and past cannabis users. Lastly, as the extracted qualitative data was not linked with individual participant data, it was not possible to examine the frequency of cannabis use specifically within those who provided a free-text response. Therefore, future work should investigate whether the terminology reported here are only preferred by a subgroup of cannabis users with particular usage patterns.

One of the key strengths of the present study was the rigorous and transparent methodological procedure used to analyse the qualitative data. This increased the contextual validity of results, especially due to the small amount of variance regarding terms mentioned and contrasting responses. Therefore, themes could be clearly coded depending on the cannabis term mentioned and easily validated through each stage. Secondly, this was one of the first studies to look into responses to terminology from the perspective of individuals who use cannabis frequently, linking to the current importance of public and patient involvement in research.

## Conclusions

In conclusion, this study underscores the importance of incorporating views of experienced individuals who frequently use cannabis on terms currently used to describe cannabis in research contexts to ensure that assessments use credible terms, connecting the researcher with users in the general population. Results presented here indicate a need to move towards descriptors that incorporate information on the concentration of key cannabinoids as well as information on methods of preparation and consumption.

## Supplementary Information

**Additional file 1: Appendix 1**. Enablers file. **Appendix 2.** Summarised analysis file. **Appendix 3.** Frequency of responses for each cannabis strain.

## Data Availability

Summarized data is available from the authors on request, and other results from this survey have been reported previously (Sami et al. 2019). The authors do not have ethical approval to provide individual participant data.

## Abbreviations

THC: Delta-9-tetrahydrocannabinol
CBD: Cannabidoil
