# Supplementary material for: Are researchers getting the terms used to denote different types of recreational cannabis right?—a user perspective
Source: J Cannabis Res. 2021 Apr 29;3:12. doi: 10.1186/s42238-021-00065-1 (PMC8086348; doi:10.1186/s42238-021-00065-1)
Supplement: Supplementary file 1 — Additional file 1: Appendix 1. Enablers file. Appendix 2. Summarised analysis file. Appendix 3. Frequency of responses for each cannabis strain. [file 42238_2021_65_MOESM1_ESM.pdf]

Appendix 1: Enablers file

1.01 Regular cannabis
Just smoke regular weed/cannabis [p1016, p457].

1.02 Oils
Generally use oil [p291,p1001,p320,p714, p834, p841,].—
Oils are effective at dealing with pain/anxiety [p115]—
Oils have better balance of CBD to THC [p115]—
Make oil from skunk or any other strain [p1143].—
There are many methods of extracting oils [637].—
Vape pen heats up cannabs oil and inhales [p808,570, p1154].—
Inhaling through vape pen better than smoke from flames [p808].—
Chocolate infused with hash oils [p110].—
Hash oil used, often known as dabs [p298].
Specifically use Butane Hash Oil [p2,p270,p422,p1153].—
Specifically use CBD oil [p427,p996].—
There are many different types of CBD oil [p996].—

1.03 Haze
General Haze consumed [p87,p441,p506,p176,p981,p697,p649,p1025,p791,p224]—
General Haze being the most common strain used [p60].—
General haze far stronger weed than super skunk [p345].—
Lemon haze consumed [p301,p889,p859,p971,p153,p28,p1065,p134,p411,p813,p813,p894,p877]
Used lemon haze recently once [p971]—
In England you mainly get super lemon haze [p301].—
Generally smoked amnesia haze [p901,p65,p411,p134, p1071,,p828,p1063,p1077].—
Generally smoked silver haze [p40].—
Generally smoked grapefruit haze [p84].—
Generally smoked super silver haze [p102,p400,p625,pp834].—
Super silver haze is a very strong strain [p625].—
Generally smoked super lemon haze, very strong strain [p625]. —
Generally smoked super bubblegum haze [p192].—
Generally smoked abynissia haze [p834].—
Generally smoked mango haze [p983].—
One of the strains available by dealer was golden haze [p593]—

1.04 Power
Pure power plant is my favourite [p1094].
Generally smokes power [p451].
Pure power plant is a strong strain [p625].

1.05 El Nino
Generally consumes El Nino [p451].

1.06 g13
Generally consumes g13 [p461,p834].

1.07 C99
Home grown C99 strain consumed [p1178].

1.08 Cali
Generally consumes Cali [p1178].

1.09 Moroccan Black
Moroccan Black was good in the mid 90’s [p1191].

1.10 Wheelchair
Generally consumes wheelchair [p451].

1.11 Sweet train wreck
Sweet train wreck is a personal favourite- makes you fee good without obvious effects majority of strains have [p451].
Gives a light head buzz [p451].

1.12. Lemon og
Generally consumes lemon og [p451].

1.13. Plushberry
Generally consumes Plushberry [p451].

1.14. Whitewalker og
Generally consumes Whitewalker og [p451].

1.15. Black russian
Generally consumes Black russian [p451].

1.16. Supersour diesel
Generally consumes Supersour diesel [p451].

1.17. Organic regs
Generally consumes Organic regs [p451].

1.18. Hash
Sometimes consumes hash [p84].
Generally consumes hash/moroccanhash [p1182,p558] .
Hash is really tasty and sedating [p301].
Consumes high quality imported hash [p390].
Doesn't smoke hash [p1068].

1.19. Sour Pink Grapefruit
Generally consumes sour pink grapefruit [p411]

1.20. Ak47
Generally consumes Ak47 [p461,p411]

1.21. Luke Skywalker
Generally consumes Luke Skywalker [p595]

1.22. White Russian
Generally consumes White Russian [p373]

1.23. Wappa
Generally consumes Wappa [p373]

1.24. Grapefruit
Generally consumes Grapefruit [p84]

1.25. Blue dream
Generally consumes Blue dream [p310, p983]

1.26. Cherry Pie
Generally consumes Cherry Pie [p310]

1.27. MK Ultra
Last strain there dealer had in the last month included MK Ultra [p593].

1.28. Spice
Period of smoke smoking almost killed them [p721].
Quit smoking this when moved from Sweden to Spain [p721].

1.29. Cinnex
Generally consumes Cinnex [p310]

1.30. Sour Diesel
Generally consumes Sour Diesel [p310, p905, p981]

1.31. Strawberry cough
Generally consumes Strawberry Cough [p378]

1.32. White widow
Generally consumes White widow [p642, p684, p554]

1.33. California Orange
Generally consumes California Orange [p1073]

1.34. Auto Fems
Auto fems grown at home [p554].

1.35. Jack Herer
Jack Herer perfect for medical and recreational purposes [p953]
Jack Herer is a indica dominant hybrid: combines shiva skunk with northern lights (indica). [p967].
Small grown producer provides it because they are ill [p967].
Generally consumes Jack Herer [p84, p134, p796]
Jack Herer helps you stay active [p102].

1.36. Pineapple
Generally consumes Pineapple [p389]

1.37. Pineapple express
Generally consumes Pineapple express [p1073]

1.38. Tangerine dream
Tangerine Dream is there favourite but can only get in Amsterdam [p859]

1.39. Bubblegum
Generally consumes Bubblegum [p134].

1.40. Skunk
Generally consumes Skunk [p1224, p717, p935].
Has used various skunk crosses [p1025].
Never has had an issue with skunk strains at all, as long as you consume responsibly like any other high THC strain [p1025].
F1 skunk is ok [p324].
High sativa skunk is preferred by them [p375].
Herbal cannabis consumed, has had skunk #1 [p790].

1.41. Northern Lights.
Generally consumes Northern Lights #5 [p324, p716, p834, p1015].
Combine shiva skunk with northern lights (both indica) to create indica dominant hybridd Jack Herer [p967].

1.42. Landrace
Generally consumes Landrace [p15].
Landrace strains are the best [p324]

1.43. Kush
Generally consumes Kush [p506, p642, p649, p791, p1025, p1063].
Started to get different kush's in the UK [p301].
Kush is a good muscle relaxant and helps as a sleep aid for back pain and constant sciatica [p1071].
Generally consumes Bubbleberry Kush [p28].
Generaly consumes Afghan Kush [p593, p40].
Generally consumes God's Gift's rapper kush [p310].
Favourite strain of cannabis that is home grown is og kush [p373].
Would like to find more og kush [p1077].
Generally consumes critical kush [p451].
Generally consumes purple kush [p603]
Generally consumes pineapple and lemon kush [p828].
Generally consumes quantum kush [p1094].

1.44. Kerala GANJA (kg).
Generally consumes Kerala Ganja [p8].

1.45. Imported in general
Imported hash and flowers [p390, p460].
Imported herbal cannabis [p1212].
Imported cannabis almost always much stronger than skunk [p1131].
Smoke marijuana homegrown by friends or imported from abroad [p1165].

1.46. Cheese
Generally consumes Cheese [p28, p1025, p1063, p1113, p57, p87, p102, p192, p697, p791, p813, p821, p978, p461].
Cheese is highly regarded to get a high [p268].
Mainly get Cheese in england/ its common [p301, p268, p376, p953
Avoids cheese for aesthetic reasons [p845].
Thinks you mean strong hybrid strains like cheese rather than skunk [p441].
Doesn't know name of strain but sometime dealer says its cheese [p340].
Cheese and blue cheese are effective and stronger than other strains as it is often sensimilla millia when homegrown [p323].
Generally consumes Blue Cheese [p40, p981].
Generally consumes Dutch Cheese [p373].
Generally consumes Buddha Cheese [p593].

1.47. Green
Generally consumes some green [p285].

1.48. By origin
Generally consumes some Moroccan Afgan''''Indian [p285].
Generally consumes some Cambodian grown [p603].
Generally consumes from Amsterdam [p859, p506, p810].
Generally consumes some Californian import grown [p810].
Grew in Caifornia with medical recommendation for years [p298].
Generally consumes cali or california orange [p461, p1073].
Generally consumes Malawi imported cannabis [p1076].
Generally consume Morocco imported cannabis [p558].
Generally consumes Moroccan black [p1191]
Generally consumes Thai weed/stick [p411, p504].
They were living in South Africa so unknown strain type [p1233].

1.49. Indica/Sativa
There are two different types of cannabs: indica and sativa [p40
Prefers hybrids over sativa/indica [p49
Mostly buys sativa but now and again gets indica [p84, p504
Consumes indica- white widow and cheese and sativa- jack here and super silver haze [p102].
Consumes indicas and sativas [p310, p1077, p1087].
Thinks researcher believes super skunk is what indica and sativas are [p476].
Indica and sativa are the right terms to use nto skunk and superskunk [p565].
Prefers sativas over indicas [p431, p964].
1:1 THC/CBD Indica strain consuemd [p780].
Smokes Indica only, mostly grown in the UK [p794].
Smokes Indica with as low THC level as possible [p922]
Sativa heavy hybrids are what they like the most, such as Jack Herer perfect for medical and recreatinal purposes [p953].
Uses Jack Herer- an indica dominant hybrid made by combining shiva skunk (indica) and northern lights (indica) [p967].
Sometimes smokes indica sometimes sativa sometimes hybrid mostly strong strains [p1067].
Indicas with good amount of CBD are excellent muscle relaxants and helps with sleep for their back pain and constant sciatica [p1071].
Consumes Cheese and Amnesia- two types of indica or sativa [p1113].
CBD dominant sativa strains throughout the day, indica dominant strains with profound antispasmodic effects at night [p1124].

1.50. Resin
Generally consumes resin [p892].

1.51. Bush weed
Generally consumes bush weed [p1183].

1.52. Soap bar
Soap bar low quality more widely available [p1191].

1.53. Hybrids
Generally consumes hybrid strains [p15, p321, p310].
Prefer hybrids over sativa/indica [p50.
Thinks you mean heavy hybrid strains instead of skunk [p441].
Theres a lack of sativa heavy hybrids which is what they like the most such as Jack Herer- perfect for medical and recreational purposes [p953].
Uses Jack Herer- an indica dominant hybrid [p967].
There are many hybrids that have far higher thc content [p992].
Smokes some sativa, some indica and some hybrid [p1067].
Uses hydroponic hybrids [p592].

1.54. Medical strains low CBD
Used to grow indoor and outdoor and California with medical recommendation [p298].
Opts for strains higher in other cannaboids and lower in psychoactive CBD [p54, p1067].
Availability of these kinds of strains are poor in this country [p54].
Consumes Bedrocan- has success in symptom relief under contract for Dutch Ministry of Health [p54].
Homegrown medical grade strains [p579].
Jack Herer- perfect for medical purposes [p953].

1.55. THC/CBD mention
Cannabis oil has better balance of CBD to THC [p1155].
Smokes high THC/high CBD strains [p1335].
Indica has fairly high THC/CBD ratio as buds are well formed and have good coating of trichomes [p84].
1:1 THC/CBD ratio indica strains [p780].

Home grown weed high THC/CBD content [p1069].
Home grown high CBD strains [p992].
Consumes 5% CBD 5% THC cannabis during day and 15-25% before bed [p108].
Consumes cannabis oil- THC wax, concentrated THC smoked off a vaporising pin [p28].
High sativa skunk preferred as has good THC level [p375].
Brick weed has low THC bad buds [p375].
Prefers as low THC levels as possible, usually going with indica [p922].
Uses a variety of strains with different effects with high THC [p413].
Saying super skunk/skunk instead of high THC level [p534, p1095, p1113, p14, p1012].
Consumes Oils and pure THC [p320].
Girl scout cookies consumes indica over 20% THC low CBD and pineapple express over 22% THC [p389].
Consumes Blue blood 10% CBD [p1025].
Likes other strong/high THC strains you need to be responsible to know the difference between use and abuse [p1025].
Consumes cannabis oil in pill form- 500mg CBD [p945].
Prefers higher rate CBD/CBD amongst other cannaboids to psychoactive THC [p323].
Less THC more CBD most effective medicinal/pain relieving properties [p323].

1.56. Herbal cannabis
Herbal vapouriser (flower mate mini)- heats cannabis below combustion point: cannabinoids/terpenes evaporate- plant matter left behind- no tar/other components- only active compounds [p84].
Uses herbal cannabis, grown organically [p450, p790, p1212].
Herbal cannabis occasionally available [p1191].

1.57. Sinsemilla/ Sensimilla seeds
Cheese and blue cheese often sinsemilla when homegrown and so quite strong [p323].
Sensimilla seeds- white widow auto fems x3 grown at home [p554].

1.58. Edibles
Occasionally has edibles [p108].
Generally has edibles [p892, p404, p458, p650].
Consumes chocolate infused with hash oils [p110].
Makes cannabis butter with already vaped herbs and coconut oil making an appetite stimulant [p967].
Tried butter weed with coffee which was relaxing [p301].
Infused coconut oil [p650].

1.59. Homegrown
Cheese and blue cheese is sinsemilla when homegrown [p323].
Used to grow they're own now buy homegrown from friends and connections [p646].
Used to grow indoor and outdoor at California with medical recommendation for years [p298]
Generally homegrown/ grown by friends [p594, p1215].
Organic grown generally [p1179].
Home grown leaf [p350].
Grows own from high CBD seeds [p281].
What is home grown to resaerchers- grown in they're own home or just not imported from abroad [p317].

1.60. Anything 'grown well'
Anything grown well is what one aims for [p366].
Will get some low grade if it grows well [p1211].

1.61. Concentrates
Cannabis oil (THC wax)- concentrated THC smoked off a vaporisingg pin [p28].
Rarely uses concentrates [p144].
Generally uses concentrates [p404].
Mostly concentrates such as shatter/wax [p460].
Either vapes medical weed or concentrates- oil/shatter/wax in vaporiser [p570].
Concentrates like shatter and crumble [p852, p957, p1101].

Wax/shatter and concentrates widely used but too much money (£40-100 per gram) to strong for them and builds dependance/tolerance levels [p1105].

1.62. High grade

Smokes high grade (which old people call skunk)- p301
Smokes high grade weed which is the umbrella term looking for not skunk [p821].
Usually smokes high grade never below mid [p1027].

1.63. Unsure

Most dealers don't have a clue whether the cannabis was imported or homegrown and fewer tell the customer [p84].
Doesnt know what they consume [p772, p984, p904, p1055, p311].
Just gets a bag with buds in and a few leaves [p984].
Shared a joint with a friend so doesn't know what they consume [p932].
Have never bought it personally only used when other people have passed around [p688].
Half the time the dealer doesn't know what strain they sell [p407].
Doesn't know whether imported or home grown [p1068].
Doesnt know where it comes from, how or where it is grown [p1095].
Has only tried it three times in life so doesn't know [p256].
Only tried it once in 1976 [p750].
Only tried on two occasions [p1230].
Doesn't know specific brands because they are incredibly difficult/rare to come across in Britain due to the illegality and how far behind we are in comparison to other countries such as the US and parts of mainland Europe [p802].
If it were legal would be able to use better strains for pain management but uses whats available [p867].
Doesn't have the luxury of choosing- anything green and strong will do [p1034].
Although specific strains are mentioned- the product rarely matches the description [p1040].
Just smokes whatever South London has to offer [p1040].
It is illegal and somewhat hard to obtain in my area [p1171].

1.64. Amnesia

Generally consumes Amnesia [p593, p1073, p1113, p939, p889].

1.65. Synthetic

Has smoked fake weed before but made them feel very unwell [p1122].

2.01 Problems with word 'skunk'

If you mean do I smoke the flower of the plant then yes but that doesnt necessarily mean that its skunk. [p11].
Not all cannabis flowers are skunk. [p11].
Maybe do some research instead of using terminology used in the media. [p11].
Secondly skunk and super skunk they find derogatory terms used to describe cannabis with low cbd content and a higher thc content [p14, p1095, p108, p84]
""Skunk"" is actually the term of a single strain of marijuana and is incredibly incorrect to use the term when referring to cannabis as a whole [p1113].
Do some research skunk is not necessarily sensimilla and there are way more strains than skunk [p57].
There are a thousand and one strains available. Skunk is one of them. It's like calling your vacuum cleaner a Hoover even if it's made by Dyson. [p317].
Skunk is a specific strain and therefore a gross generalisation [p324, p425, p513, p621, p821, p974, p992, p1011, p1074, p380, p1012, p1113, p84, p1143].
Using word skunk promotes hate against cannabis [p425].
Indica/sativa are the right terms skunk is just one strain [p565, p964].
Skunk is herbal cannabis,why the separation? [p560].
Skunk is 20 year old strain not really used anymore. [p577].
It''s a load of rubbish when people say skunk is a stronger weed. [p621].
Also, using the tabloidism 'skunk' as a category is about as logical as calling every dog a Labrador. [p974].
Please stop using skunk as a word other than a strain. Just say low strength (5-10%) mid strength (10-18%) and strong (18+%) [p1084].

Cannabis users use the word skunk to describe certain types of cannabis that have a particular aroma, has nothing to do with strength.
[p1131].
Term Skunk is overused and incorrect [p1067, p967].
Skunk is a misleading media term talking about high levels of THC slow CBD- skunk not actually strong at all [p1012].
Skunk- used in tabloids shouldn't in scientific surveys [p786, p790, p1135, p1096, p441, p347, p777, p263, p273, p279, p458, p264].
Should say weed not skunk [p1044].
Using the word skunk as a buzzword causes us to lean heavily away from the term [p1096].
Skunk is a myth/doesn't exist/don't know what it is [p457, p560, p472, p961, p1165, p1089, p100].
Word skunk rarely used/outdated [p60, p301, 545, p577].
Skunk is herbal cannabis, shouldn't be separated [p560].

2.02 Problems with word 'super skunk'
Doesn't know what super skunk is [p11, p413, p784, p565, p84, p432, p441, p1018, p1128, p1131, p1133, p1165].
Super skunk is just a specific strain there are other strains [p380, p414, p324, p577, p794, p604, p1113, p1067, p1084, p783, p986, p534, p50, p947, p1113, p84].
Super skunk made by crossbreeding skunk and afghani male which improves yields not strength [p784].
Doesn't refer to a very strong strain as you seem to think [p794].
Using low to high strength would be better for multiple choice than this terminology [p604].
Shouldn't use it to generalise high THC/low CBD [p84, p113, p534, p1113, p14, p84].
Do you mean indica/sativa instead? [p476, p964].
Super skunk doesn't exist [p279, p427, p273, p460, p472, p57, p84, p1089].
Super skunk is a propaganda/media term [p458, p845, p540, p458, p428, p1090].
Question using these terms are idiotic and uninformed [p485].
Can't tell difference between homegrown and super skunk [p1111].
Super skunk refers to more common homegrown high strength cannabis [p1111].
Can't tell difference between skunk and super skunk [p423, p560].
May use this to talk about strong weed but people use less in that case [p460].
Don't know much about the subject if using this term [p777, p786, p1060, p488].
Hasbeen smoking for over 10 years and couldn't confidently tell you the difference between your imported herbal, home grown and super skunk - which is the problem with drug policy [p1107].
Classed super skunk as same as home grown [p278, p803].
Super skunk can be homegrown or imported in [p375].
Super skunk is an outdated term [p791].

2.03. Problem with word 'imported'
Imported thing of the past in the UK [p14].
Less imported in the UK especially in past few months due to rapid rise in people growing their own [p545].
Unknown whether cannabis is imported/homegrown by dealer or customer [p84].
Can't tell the difference between homegrown or imported herbal which is the problem with drug policy [p1107].

2.04. Problem with word 'homegrown'
Super skunk can be homegrown or imported in [p375].
Homegrown is anything grown at home doesn't matter what strain it is [p794].
Don't think researchers now what there term homegrown means [p794].
Homegrown is something different to sensimilla, they are plants grown at home without the help of UV light and other equipment [p986].
Not sure of the distinction between homegrown and super skunk [p1111].
Sensimilla isn't homegrown skunk its cannabis grown without seeds being produced by the growing plant [p425].

2.05. Problem with word 'sensimilla'
Sensimilla, simply means without seeds and is not a type of skunk [p604].

Sensimilla is simply Marijuana without seeds [p986, p1067, p1131].
Sensimilla doesn't indicate quality at all [p1131].
Sensimilla is just a term for unfertilised female cannabis plants [p1143].
Sensimilla is an outdated term [p458].

2.06. Problem with word 'synthetic'
Synthetic only created because there was no regulated market [p432].
Would never contemplate using it [p432, p1113, p323].
Shouldn't be considered cannabis at all [p340].
Only used because it was easier to get than cannabis but never again [p340].
Totally irrelevant to cannabis [p500].
Don't imply its he same as cannnabis [p534].
Using this term can be insulting to a lot of people [p832].

2.07. Problem with word 'spice'- irrelevant/ just to market synthetic drug
Totally irrelevant to cannabis [p500].
This term gives cannabis a bad name [p1031].
Name use just sosomebody who was really desperate to market their new synthetic drug [p500].

2.08. Generally not adequate/enough options
Vaping was not an option [p967].
Vaping is a safer and more efficient way of consuming [p967].
No vaporising or dabbing as method of consumption [p485, p108].
Type list quite limited [p593, p40].

2.09. Alternate methods of categorising
Type options just trying to show how evil/scary smoking is [p804].
Suggest category of flower and concentrates- only ones needed [p832].
Just general groupings apart from the 4th option which is a specific strain [p82].

2.10. Too many strains to name
Too many strains to name as used to grow indoor and outdoor [p298].
Many different strains, cannot specify [p644, p450].

2.11. Questions are leading-bias conclusions
Definition of cannabis types are incorrect and misleading [p380, p108].
Will result in bias conclusions due to leading questions [p108].
Using inaccurate terms from misleading media sources to talk about high levels of THC and low CBD [p1012].

3. 01 Alternate way consumed
Herbal vapouriser (flowermate mini)- heats cannabis below combustion point: cannabinoids/terpenes evaporate- plant matter left behind- no tar/other components- only active compounds. [p84].
Vapouriser is primary way of consumption [p108].
Vaporising or dabbing method of consumption option not shown [p485, p967, p10132].
No vaporising option means survey is incomplete [p570].
No menotion of concentrates e.g. i.e. Rosin tech, BHO, QWISO, or QWET, or Vaporizing [p974].
Uses a MediPen CBD vapouriser when travelling which is intrinsic when they do not have access. [p1124].
Vaporises rosin or solventless hash oil [p1154].

4. Information about the sources for strains
Url given: http://www.mrnice.nl/forum/5-strain-base/ [p791].
URL given: https://www.leafly.com/indica/super-skunk https://www.leafly.com/indica/afghan [p380].
Source of strain- needs to be grown and cured properly otherwise like moly fruit its as good as poison [p625].
Criminals are in charge of this process for 3.2 million citizens [p625].

Appendix 2: Summarised analysis file

1.01 Regular cannabis
Only two participants stated they smoked 'regular' cannabis, which other participants were more specific with their responses on cannabis consumed.

1.02 Oils
After the statements of the general consumption of 'oil' and specifically 'butane hash oil', the next most common responses relating to this category was the use of a vape pen heating up the cannabis oil before it is inhaled. One participant stated that this was better than smoking from flames, suggesting its need to be considered in the 'methods of consumption' question. Furthermore, participants mentioned that there are many methods of extracting oils and oils can be made from skunk or other strains. Therefore, there are many different types of oils, further enforced by the different oils mentioned such as chocolate infused hash oil compared to different CBD oils. Specificity is needed when speaking about oil consumption. Lastly, different terms can be used when speaking about oil consumption, such as 'dabs' that should be considered.

1.03 Haze
Lemon haze was the most popular haze strain consumed, mentioned by more participants than general mention of 'haze'. Amnesia haze was the next most possible haze strain mentioned, before super silver haze and less popular strains such as grapefruit, abynissia, mango, silver haze, super bubblegum haze and golden haze. In terms of strength, participants mentioned general haze to be far stronger weed than other strains such as super skunk, specifically mentioning super silver haze and super lemon haze. When mentioning popularity, a few participants mentioned general haze to be the most common strain used, one specifically mentioning super lemon haze. Therefore, it could be summarised that the lemon haze strain is the most popular in relation to general haze strains.

1.04 Power
This category was only mentioned by three different participants, one stating that they general smoke 'power', while the other two stating it is there favourite and a strong strain. However, it cannot be considered popular compared to other types of cannabis mentioned in this category.

1.05 El Nino/1.06 g13/1.07 C99/1.08 Cali/1.09 Moroccan Black/1.10 Wheelchair
Only a few participants mentioned these strains. One participant mentioned generally consuming El Nino, Cali, Wheelchair and homegrown C99, with only two consuming g13. Moroccan Black was stated to be a good strain in the mid 90's, suggesting that it may be an outdated strain not consumed nowadays.

1.11 Sweet train wreck
A participant mentioned that it gave a light head buzz while another stated it was a personal favourite, as it makes you 'feel good' without obvious effects majority of strains give. This could suggest that this strain provides a milder effect.

1.12. Lemon og/1.13. Plushberry/1.14. Whitewalker og/1.15. Black russian /1.16. Supersour diesel/1.17. Organic regs
Only one participant for each of these categories stated that they generally consume this strain type. This suggests that if they are not popular strains, they should not each be given an option in the 'type of cannabis' question.

1.18 Hash
Participants stated to general consume hash or moroccan hash, with a few participants stating that it is really tasty and sedating. One participant specifically stated that they consume high quality imported hash, while some raised hash as either being sometimes or never consumed. This suggests that while it may be more popular than other strains mentioned by one participant, it could be specifically raised as a strain not consumed, meaning it is not always everyones preference.

1.19. Sour Pink Grapefruit/1.20. Ak47/1.21. Luke Skywalker/1.22. White Russian/1.23. Wappa/1.24. Grapefruit/1.25. Blue dream/1.26. Cherry Pie/ 1.27 MK Ultra

Apart from Ak47 and Blue dream which were generally mentioned by two participants, the rest of the strains were raised as a strain generally consumed by one participant each. MK Ultra was only mentioned as a strain the dealer was selling, suggesting that the participant may not have actually consumed it themselves. Therefore, this information emphasises the variety of strains that could be mentioned, with only a few participants mentioning each strain type. This questions how specific categories should be classified as relating to types of consumption.

1.28 Spice
Only mentioned by one participant who said that it almost killed them, causing them to quit smoking it when changing country. This suggests that it is not even considered by other participants and therefore may not need to be mentioned as a category of cannabis consumption type.

1.29. Cinnex/1.30. Sour Diesel/1.31. Strawberry cough/1.32. White widow/1.33. California Orange/1.34. Auto Fems
White Widow and Sour Diesel were mentioned as being generally consumed by three participants, while the other strain types only by one participant each.

1.35. Jack Herer
Six participants generally consume this strain, that was mentioned to be an indicate dominant hybrid, combining shiva skunk with northern lights indica strain. This suggests that hybrid strains could be mentioned as a separate category, incorporating the possibility of strains being combined together. It was also stated to be perfect for medical and recreational purposes that also helped you stay active. One participant stated that it is provided by a small grown producer due to there clients being ill, suggesting that it may be used more to reduce pain.

1.36. Pineapple/1.37. Pineapple express/1.38. Tangerine dream /1.39. Bubblegum
One participant for each of these categories stated that they generally consumed these strains. Tangerine strain specifically from Amsterdam, while the other strain locations were not raised.

1.40 Skunk
Skunk was mentioned in different ways; skunk in general, F1 skunk, skunk #1, high sativa skunk as well as different skunk crosses. 'Skunk' may therefore be too general a definition to categorise cannabis types. One participant stated that skunk needed to be consumed responsibly like any other high THC strain so that no health issues would arise.

1.41 Northern Lights
Four participants stated that participants generally consumed this strain, while another consumes Jack Herer; a combination of this strain with shiva skunk.

1.42 Landrace
Two participants mentioned consuming this strain, with one stating it was there preferred cannabis type.

1.43 Kush
Generally mention of Kush was the most popular response, with all other responses being briefly mentioned by one participant each. It was stated to be a good muscle relaxant, aiding sleep when suffering from back pain and constant sciatica. One participant's statement that you can get different Kush's in the UK supported the mention of different Kush strains by different participants, namely Bubbleberry, Afghan, God's Gift rapper, og, critical, purple, pineapple, lemon and quantum Kush. Therefore, the use of the general Kush term can reliably group different strains together.

1.44 Kerala GANJA (kg)
One participant generally mentioned consuming this strain type.

1.45 Imported in general
Imported hash, flowers and herbal cannabis was mentioned, with another participant stating that imported cannabis was almost always much stronger than skunk. Another participants mention of both cannabis

imported from abroad as well as homegrown by friends shows no preference for imported, suggesting its lack of popularity due to importation only being mentioned by five participants.

1.46 Cheese
Generally consumption of cheese was a very popular response, with other participants stating it is highly regarded to get a high and is mainly common in England. This would explain why one participant doesn't know what strain they get unless dealer tells them its cheese, suggesting that it could be commonly sold by dealers. However, this cannot be assumed by one participants response. Different variety of cheeses were mentioned, showing how cheese also groups a variety of different strains (such as blue, dutch and buddha cheese). In terms of strength, cheese and blue cheese were mentioned as being stronger than other strains, that may be due to it being 'sensimilla when homegrown'. However, one participant states that they avoid these for 'aesthetic reasons'. This suggests that reasons for not consuming specific strains could also be an interesting point as well as strains and cannabis types that are consumed.

1.47 Green
One participant generally mentioned consuming 'green'.

1.48 By origin
Many different areas mentioned, most popular being Amsterdam, probably due to cannabis being legal in the Netherlands. Californian import was the second most popular response, which one participant stated was grown with medical recommendation for years. Other areas include Morocco, Malawi, Thailand, South Africa and Cambodia. It is important to distinguish between cannabis that was imported from another origin compared to cannabis consumed in this area, such as living in South Africa and consuming cannabis compared to living in England and having it imported from there. However, if origin is not seen as that important then specificity of this kind may not be required.

1.49 Indica/Sativa
Most of these responses suggest that both indica and sativa are consumed for different reasons. One participant smokes sativa strains during day while indica during night due to their antispasmodic effects. This was also emphasised by another participants preference for indica with a good amount of CBD, stating that it acts as a excellent muscle relaxant and help with sleep for back pain and sciatica. However, other participants smoke both for no particular reason other than preference to different strains in both categories, such as cheese and amnesia. When a preference for one is mentioned, it is usually for indica. Participants have mentioned that indica with low THC levels are preferred, just having a general preference, or due to the muscle relaxant properties previously mentioned. The two participants who preferred sativa did not give a reason for their preference. Hybrids were also mentioned in this category, specifically using Jack Herer as an example, emphasising the importance as having hybrids as a category of cannabis type. Lastly, participants did mention that indica and sativa categories are preferred to skunk and super skunk terms, that should be taken into account.

1.50 Resin/1.51 Bush Weed/1.52 Soap Bar
One participant mentioned each of these categories as a type of cannabis they consumed, while Soap Bars were stated to be consumed due to being widely available. However, that fact that it was only mentioned by one participant suggests that other types are a lot more popular despite there supposed availability.

1.53 Hybrids
Specific mention of Jack Herer suggest that it is a popular hybrid consumed by participants. One participant stated that this hybrid was perfect for medical and recreational purposes, suggesting why it is popular among participants. Participants also stated that they consumed it generally as well as indica and sativa while another participant stated a preference over hybrids specifically. Mention of hydroponic hybrids should also be considered, as well as another participant statement that hybrids that have far higher thc content are often appealing to participants.

1.54 Medical strains low CBD
Once again, Jack Herer has been mentioned specifically for medical purposes, emphasising its popularity of use for a variety of reasons. Furthermore, California has also been mentioned again as an origin to grow cannabis with medical recommendation. A few participants mentioned the use of strains lower in psychoactive CBD and

higher in other cannaboids, that may not be that available in the participants country. However, one participants mention of higher CBD than THC as being more effective for pain relief does suggest some controversy. Also, Bendrocan was specifically mentioned as a successful method of symptom relief.

1.55 THC/CBD mention
Participants have specifically mentioned the ratio of THC/CBD, such as consuming 5% of both during day and 15-25% before bed or mentioning strains with specific measurements, like blue blood (10% CBD), pior girl scout cookies (20% THC). A popular statement raised by five participants suggest that super skunk and skunk terms used should instead be called cannabis with high THC levels, questioning whether the use of these terms that will be discussed later on. One participant also stated that you need to be responsible enough to know the difference between use and abuse when using high THC strains. This suggest that there is some controversy on whether higher THC or CBD is better, supported by the fact that some participants preferred 1:1 ratio, others preferred higher CBD or THC and some showed no preference to either at all. Participants also mentioned cannabis oil, stating it has a 'better balance of CBD to THC', mentioning its use for THC wax (concentrated THC smoked off a vaporising pin) and use in pill form (500mg CBD). Other mentions include the use of brick weed, that has low THC buds, high sativa skunk with good levels of THC and indica with low THC levels. While there is a lot of contrasting opinion on what THC/CBD level is preferred, the general mention of these term suggest that it is an important mention of categorisation.

1.56 Herbal cannabis
As well as the general mention of organically grown herbal cannabis which is occasionally available, the mention of consuming herbal cannabis using a flower mate mini vaporiser suggests that other methods of consuming cannabis need to be included in the survey.

1.57 Sinsemilla/Sensimilla seeds
The mention of this category by two participants questions its insertion as another option for cannabis type use, even though it is mentioned. One participant mentioned sinsemilla in regards to cheese and blue cheese while another mentioned sensimilla seeds when speaking of white widow auto fems x3 grown at home.

1.58 Edibles
Generally mention of edibles was the most popular response, while the other responses suggested the variety of edibles that are available for participants. This included infused coconut oil (seen as an appetite stimulant), butter weed with coffee (seen as relaxing), cannabis butter with already vaped herbs and chocolate infused with hash oils. The different variety could possible considered when mentioning the generally 'edibles' category depending on its importance to the researcher in future survey updates.

1.59 Homegrown
Participants generally mentioned growing their own organically, home grown leaf or from high CBD seeds or consuming cannabis grown by friends. One participant spoke of cheese and blue cheese being sinsemilla when homegrown while another was unsure about the term homegrown: grown in their own home or just not imported from abroad. Perhaps this term needs to be better explained, although only one participant made this comment.

1.60 Anything 'grown well'
Two participants stated that they would get anything 'grown well' even if it was low grade.

1.61 Concentrates
Many participants spoke of generally consuming concentrates such as shatter, crumble and wax, which were not included in the options given. One participant stated that these are too strong and can build tolerance levels, however other participants said that is what they mainly use. Cannabis oil which is THC wax was stated by one participant to be smoked off a vaporising pin, emphasising the need to use this method of consumption in the option criteria for the way of consumption question.

1.62 High grade
Two participants stated that high grade weed is a better umbrella term compared to skunk, again emphasising the importance of not using this term for future survey updates. Another participant stated that they smoke high grade not below mid grade.

1.63 Unsure
Many participants are unsure what they consume but for a variety of reasons. These include sharing a joint with friend so not buying it themselves, only trying it on a few occasions, don't know brands as they are rare in Britain due to illegality, uses whatever is available or just simply don't have the luxury of choosing. Other participants stated that they just get a bag with buds in and a few leaves but don't know the specific brands or knowing the strain without knowing whether it homegrown or imported.

1.64 Amnesia
Five participants stated that they generally consume Amnesia.

1.65 Synthetic
One participant mentioned this, stating that they smoked 'fake weed' before but made them feel very unwell.

2.01 Problem with word 'skunk'
This is the most popular category, emphasising the importance of altering or not using this term in the category. The most popular themes mentioned relate to the word skunk were that it is a tabloid/media term not used in scientific surveys, being a gross generalisation of cannabis as it is a specific strain that is rarely used by consumers and yet is overused as a term and outdated. This is the main point seen in all the themes relating to the word 'skunk'; that it is not an umbrella term but one for a specific strain like lemon haze would be. One participant believes this kind of term could even raise hate towards cannabis use. Many believe the term is a myth or doesn't exist or others plainly don't even know what the term means, probably due to its specificity as a strain.
Specifically, some participants believe that the derogatory term is used to describe cannabis with low cbd content and a higher thc content. In terms of skunk compared to other terms used in the survey, a few participants have stated that skunk is herbal cannabis and shouldn't be separated, researchers should use the word weed not skunk and that skunk is not necessarily sinsemilla. Other issues raised by some participants relate to the false pretences the word 'skunk' can pertain. Skunk may wrongly be assumed to be a 'stronger weed' type or relate to a specific aroma. Furthermore, participants state that not all cannabis flowers or sinsemilla are skunk. Some participants thought of other ways to define the terms for the survey, such as using the terms indica/sativa or low strength, mid strength and strong strength instead of skunk.

2.02 Problem with word 'super skunk'
Super skunk was referred to as one participant as a specific strain made by crossbreeding skunk and afghani male, improving yields but not strength of the cannabis. The majority of responses referred to not knowing what the term means, showing that it is not a clearly defined and well known term even to participants who regularly consume cannabis. Another recurrent statement similarly to the problem raised by skunk is that it is a specific strain, not a term to categorise a general cannabis type. It is also described as a propaganda/media term which reflects bad knowledge of the cannabis subject, some participants even stating the outdated term doesn't even exist. Participants also stated they could not tell the difference between skunk and super skunk or homegrown and super skunk. Other participants followed this response by stating that they class super skunk as homegrown and that it could be homegrown or imported. Other participants also stated it should not be used to generalise high THC/low CBD cannabis or 'strong weed'. Opinions on alternative grouping methods include low/high strength and indica/sativa, as said in 'skunk' categorical statements earlier.

2.03 Problem with word 'imported'
Compare to super skunk and skunk, the word imported and other terms to be mentioned were much less disputed, yet the complaints should still be taken into account. One participant stated that they cannot tell the difference between homegrown or imported, while another stated they as well as the dealer cannot tell whether it is imported or not. This was also raised to be an outdated term, another participant stating this was because of a rapid rise in people growing their own. Therefore, perhaps this term needs to be defined further or an option stating 'unknown' should be made available.

2.04 Problem with word 'homegrown'
Many participants stated that the term homegrown and sinsemilla are not the same and therefore should not be grouped together. Sinsemilla is simply grown without seeds while homegrown is grown without UV light and other equipment. Furthermore, regardless of strain it could be homegrown, supported by another

participants stating that super skunk can be homegrown or imported in. Therefore, it is imported to understand that the term homegrown can relate to other options available in the criteria.

2.05 Problem with word 'sensimilla'
Problems raised of this term suggest that it is outdated, simply a term for unfertilised female cannabis without seeds, not relating to quality or being a specific type of skunk at all. Therefore, it is important to think about whether this should be a grouping method, as different types could also be 'sensimilla'.

2.06 Problem with word 'synthetic'
Many participants would never contemplate using synthetic cannabis, considering it irrelevant to cannabis consumption and perhaps even insulting to participants. Therefore, if it is important to mention it in the survey, it would be better to separate it more from the other cannabis forms, not grouping it in the same category as non synthetic cannabis. Participants who do speak of its use state that they only used it because it as easier to get and would never do so again or because there was no regulated market for non synthetic. Therefore, it seems that the few who do use it consider it a last resort.

2.07 Problem with the word 'spice'
As synthetic, this term is seen as irrelevant and insulting, giving cannabis a bad name. One participant stated that it was only used to market 'someones new synthetic drug', suggesting that its use in the survey may not be warranted by participants.

2.08 Generally not adequate/enough options
As stated earlier, methods of consumption such as vaping or dabbing are not included. A couple of participants also stated that the type list is quite limited, suggesting that this must also be reviewed.

2.09 Alternate methods of categorising
Participants disagree with the specific strain use as a category type when the other options are general groupings. One participant believes that only flower and concentrates are needed as suggested categories, while another believes that the options shown just make smoking seem 'evil and scary'. Therefore, when the category options are reviewed, it is important to make sure they do not give cannabis a bad name or insult the participants, such as removing the term 'skunk'.

2.10 Too many strains to name
Grouping the different strains will be a hard task, as so many are available and therefore specification is difficult. It is important to review how important knowing the specific strain is before it should be considered in the grouping methods for category of cannabis type consumed.

2.11 Questions are leading-bias conclusions
Participants have specifically stated that the definition of cannabis types given are misleading, resulting in bias conclusions perhaps due to the terms coming from media and tabloid articles.

3.01 Alternate way consumed
Herbal vaporiser (flower mate mini), general vaporising and dabbing method should be mentioned. Furthermore, no mention of concentrates such as Rosin tech or other vaporiser forms such as MediPen. It is important to include these.

4. Information about the sources for strains
Many different urls were provided, however these would not have been if participants felt the terms mentioned were correct and well defined: a good representation of the different cannabis types. Once the survey has been revised, it could be assumed that less information sources would be given, as participants would not feel that they are required.

Appendix 3: Frequency of responses for each cannabis strain.

Inductive code	Frequency
1.01	2
1.02	11
1.02a	1
1.02b	3
1.02c	4
1.02d	2
1.02e	1
1.3	12
1.03a	12
1.03b	8
1.03c	2
1.03d	1
1.03e	4
1.03f	1
1.03g	1
1.03h	2
1.03i	1
1.03j	1
1.03k	1
1.04	3
1.05	1
1.06	2
1.07	1
1.08	1
1.09	1
1.1	1
1.11	1

Inductive code	Frequency
1.12	1
1.13	1
1.14	1
1.15	1
1.16	1
1.17	1
1.18a	4
1.18b	1
1.19	1
1.2	2
1.21	1
1.22	1
1.23	1
1.24	1
1.25	2
1.26	1
1.27	1
1.28	1
1.29	1
1.3	3
1.31	1
1.32	3
1.33	1
1.34	1
1.35	6
1.36	1
1.37	1
1.38	1
1.39	1

Inductive code	Frequency
1.40a	4
1.40b	1
1.40c	1
1.40d	1
1.41	6
1.42	2
1.43a	8
1.43b	1
1.43c	2
1.43d	1
1.43e	2
1.43f	1
1.43g	1
1.43h	1
1.43i	1
1.43j	1
1.44	1
1.45	5
1.46a	22
1.46b	3
1.46c	1
1.46d	1
1.47	1
1.48a	1
1.48b	1
1.48c	3
1.48d	4
1.48e	1
1.48f	1

Inductive code	Frequency
1.48g	2
1.48h	2
1.48i	1
1.49	22
1.5	1
1.51	1
1.52	1
1.53a	9
1.53b	1
1.54	5
1.55a	6
1.55b	1
1.55c	1
1.55d	8
1.55e	4
1.55f	1
1.55g	1
1.56	5
1.57	2
1.58a	4
1.58b	1
1.58c	2
1.58d	1
1.59a	7
1.59b	1
1.59c	1
1.6	2
1.61	8
1.62	3

Inductive code	Frequency
1.63a	11
1.63b	3
1.63c	1
1.63d	4
1.64	1
1.65	1
2.01a	1
2.01b	3
2.01c	22
2.01d	20
2.01e	7
2.01f	5
2.01g	2
2.01h	1
2.02a	22
2.02b	9
2.02c	3
2.02d	21
2.02e	3
2.02f	1
2.03a	3
2.03b	2
2.04a	2
2.04b	1
2.05a	6
2.05b	1
2.06a	1
2.06b	3
2.06c	4

Inductive code	Frequency
2.07	2
2.08	5
2.09	3
2.1	3
2.11	3
3.01	8
4.01	2
4.02	1
